# Developing and Evaluating an AI-Based Computer-Aided Diagnosis System for Retinal Disease: Diagnostic Study for Central Serous Chorioretinopathy

**DOI:** 10.2196/48142

**Published:** 2023-11-29

**Authors:** Jeewoo Yoon, Jinyoung Han, Junseo Ko, Seong Choi, Ji In Park, Joon Seo Hwang, Jeong Mo Han, Daniel Duck-Jin Hwang

**Affiliations:** 1 Department of Applied Artificial Intelligence Sungkyunkwan University Seoul Republic of Korea; 2 Raondata Seoul Republic of Korea; 3 Department of Human-Artificial Intelligence Interaction Sungkyunkwan University Seoul Republic of Korea; 4 Department of Medicine Kangwon National University School of Medicine Kangwon National University Hospital Chuncheon Republic of Korea; 5 Seoul Plus Eye Clinic Seoul Republic of Korea; 6 Seoul Bombit Eye Clinic Sejong Republic of Korea; 7 Department of Ophthalmology Hangil Eye Hospital Incheon Republic of Korea; 8 Lux Mind Incheon Republic of Korea

**Keywords:** computer aided diagnosis, ophthalmology, deep learning, artificial intelligence, computer vision, imaging informatics, retinal disease, central serous chorioretinopathy, diagnostic study

## Abstract

**Background:**

Although previous research has made substantial progress in developing high-performance artificial intelligence (AI)–based computer-aided diagnosis (AI-CAD) systems in various medical domains, little attention has been paid to developing and evaluating AI-CAD system in ophthalmology, particularly for diagnosing retinal diseases using optical coherence tomography (OCT) images.

**Objective:**

This diagnostic study aimed to determine the usefulness of a proposed AI-CAD system in assisting ophthalmologists with the diagnosis of central serous chorioretinopathy (CSC), which is known to be difficult to diagnose, using OCT images.

**Methods:**

For the training and evaluation of the proposed deep learning model, 1693 OCT images were collected and annotated. The data set included 929 and 764 cases of acute and chronic CSC, respectively. In total, 66 ophthalmologists (2 groups: 36 retina and 30 nonretina specialists) participated in the observer performance test. To evaluate the deep learning algorithm used in the proposed AI-CAD system, the training, validation, and test sets were split in an 8:1:1 ratio. Further, 100 randomly sampled OCT images from the test set were used for the observer performance test, and the participants were instructed to select a CSC subtype for each of these images. Each image was provided under different conditions: (1) without AI assistance, (2) with AI assistance with a probability score, and (3) with AI assistance with a probability score and visual evidence heatmap. The sensitivity, specificity, and area under the receiver operating characteristic curve were used to measure the diagnostic performance of the model and ophthalmologists.

**Results:**

The proposed system achieved a high detection performance (99% of the area under the curve) for CSC, outperforming the 66 ophthalmologists who participated in the observer performance test. In both groups, ophthalmologists with the support of AI assistance with a probability score and visual evidence heatmap achieved the highest mean diagnostic performance compared with that of those subjected to other conditions (without AI assistance or with AI assistance with a probability score). Nonretina specialists achieved expert-level diagnostic performance with the support of the proposed AI-CAD system.

**Conclusions:**

Our proposed AI-CAD system improved the diagnosis of CSC by ophthalmologists, which may support decision-making regarding retinal disease detection and alleviate the workload of ophthalmologists.

## Introduction

Computer-aided diagnosis (CAD) is a software system that assists in the diagnostic decision-making of clinicians [1]. CAD systems can be used to support clinicians in various tasks, such as detecting breast cancer [2], lung cancer [3], colorectal cancer [4], and even Alzheimer disease [5]. Thus, these systems potentially alleviate the heavy workload of clinicians, resulting in the improved quality of clinical services [6,7].

With recent advancements in computer vision and deep learning techniques, deep neural networks have been reported to achieve expert-level performance in clinical diagnoses [8-10]. This, in turn, has led researchers to construct CAD systems involving artificial intelligence (AI) models, such as AI-based computer-aided diagnosis (AI-CAD), to assist with clinical diagnosis, for example, by detecting major thoracic diseases on chest radiographs [6] and classifying skin cancer using skin photographs [11]. Although prior studies have made valuable progress in developing high-performance AI-CAD systems in various medical domains, minimal attention has been focused on developing and evaluating AI-CAD systems in ophthalmology, especially for the diagnosis of retinal diseases using optical coherence tomography (OCT) images.

Following age-related macular degeneration (AMD), diabetic retinopathy, and branch retinal vein occlusion, central serous chorioretinopathy (CSC) is the fourth most prevalent vision-threatening retinopathy and is characterized by serous detachment of the neurosensory retina at the posterior pole [8,12]. Most patients with CSC are male, and they experience decreased or distorted vision with altered color sensitivity and persistent subretinal fluid (SRF) damage to the retinal outer layer, resulting in permanent vision loss, which degrades their quality of life [13,14]. When diagnosing CSC, assessing the chronicity of the disease is difficult but critical for the formulation of a treatment strategy or the prediction of its prognosis [8,15]. A patient with chronic CSC with or without sustained sensory retinal detachment may already have irreversible poor vision or require active intervention; hence, preventing permanent visual disturbance that can reduce a patient’s quality of life [15] is important.

In ophthalmology, OCT is a noninvasive, rapid, and accurate test that produces highly reproducible outcomes [8,16,17]. It is frequently used to evaluate structural abnormalities associated with retinal disease, including CSC, without requiring physical contact [17]. It is now considered the imaging modality of choice for the diagnosis and follow-up of patients with CSC [18,19]. OCT has been used to examine the alterations in CSC's retinal pigment epithelium (RPE) and outer retina morphology [20]. Further, OCT can assess and quantify the presence of SRF, which can aid in estimating the episode duration and determine the subsequent treatment [17].

Herein, we propose an AI-CAD system that can alleviate the heavy workloads and improve the diagnostic performance of retinal disease for ophthalmologists. We tried to find out whether AI could really help ophthalmologists’ diagnostic activities through a CAD system in the field of ophthalmology, and we selected CSC, one of the representative macular diseases, and built a CAD system. In particular, the proposed AI-CAD system may support ophthalmologists in distinguishing the subtypes of CSC. To investigate the effectiveness of the proposed system, we conducted a within-subject user study involving 66 ophthalmologists.

## Methods

### Ethical Considerations

This study was conducted in accordance with the 1964 Declaration of Helsinki guidelines. The Ethics Committee of Hangil Eye Hospital approved the research protocol (IRB 21018) and its implementation and waived the requirement for informed consent as this study was retrospective and observational in nature and used medical records to extract the required data.

### AI-CAD System Construction for CSC-Subtype Detection

#### Data Collection and CSC Labeling

To train and evaluate the proposed deep learning model, 1693 OCT images of patients who visited Hangil Eye Hospital between June 2017 and June 2021 were collected and annotated. This study aimed to construct an AI-CAD system that identifies CSC subtypes.

All CSC cases were diagnosed by independent retinal specialists using fundus examinations, fluorescein angiography (FA), indocyanine green angiography (ICGA), and OCT images. On all CSC cases, FA and ICGA were performed simultaneously using a Heidelberg Retina Angiograph (Heidelberg Engineering) confocal scanning laser ophthalmoscope. Other potentially conflicting retinal pathologies such as AMD, polypoidal choroidal vasculopathy, pachychoroid neovasculopathy, and pachychoroid pigment epitheliopathy were excluded from our analysis.

Acute CSC was diagnosed based on the presence of serous retinal detachment involving the macula, as demonstrated by OCT, and the leakage at the level of the RPE on FA [16,17,21]. In the acute CSC cohort, only classic, acute CSC with a symptom duration of less than 4 months since the first episode was included. Chronic CSC was diagnosed based on the RPE status and was defined as chronic chorioretinopathy with widespread RPE decompensation, with or without subretinal detachment, and with or without an active leakage site, according to the Daruich et al [22] classification scheme [21]. Chronic CSC was diagnosed when extensive RPE atrophy was observed, independently of SRF, according to their definition [15,22]. Further, 2 retina experts (JSH and DDJH) reviewed the images from OCT, FA, and ICGA imaging techniques and also assessed the medical records. If there was a difference in opinions, another retina expert (JMH) stepped in to identify the inconsistency and consulted with the others. Any differences were settled through mutual agreement. Representative CSC cases are illustrated in Multimedia Appendix 1.

#### User Interface of AI-CAD System

**Figure 1:**
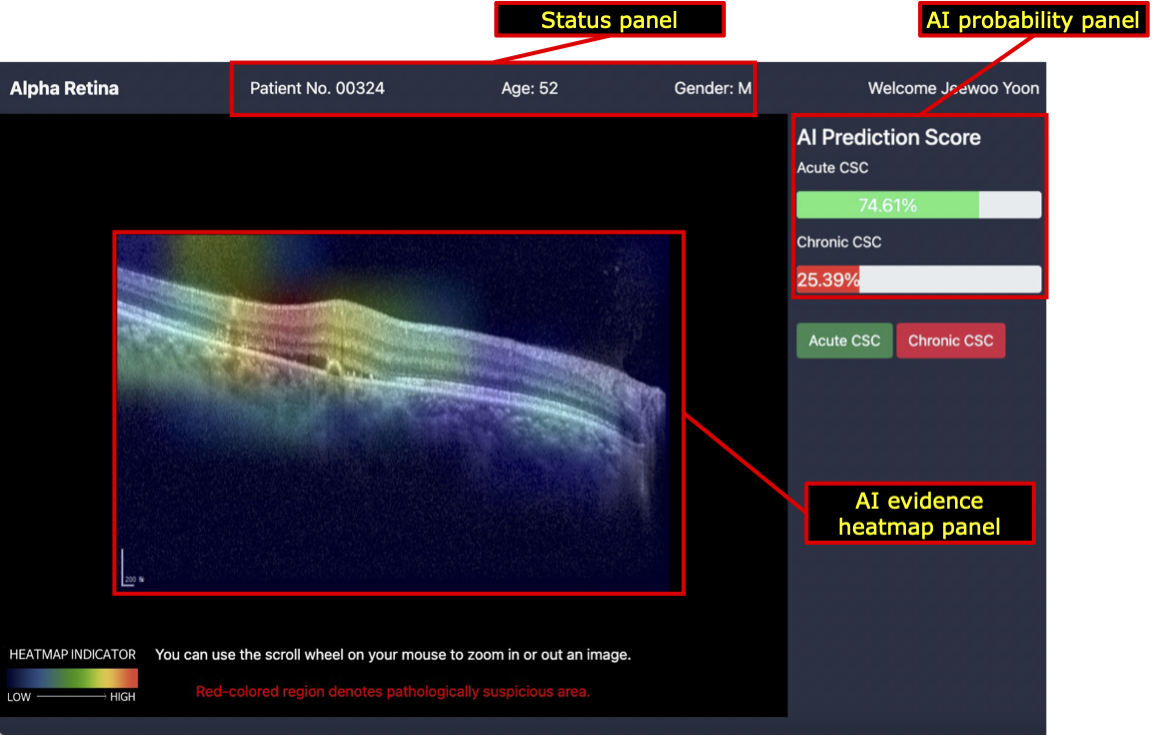
An illustration of the proposed AI-CAD system. (A) AI probability panel, (B) AI evidence heatmap panel, and (C) status panel. AI: artificial intelligence; AI-CAD: artificial intelligence–based computer-aided diagnosis; CSC: central serous chorioretinopathy; M: male.

The proposed AI-CAD system formulated in this study (Figure 1) comprises three components: (1) an AI probability panel, (2) an evidence heatmap panel, and (3) a status panel. Further, we designed the user interface of the proposed AI-CAD system using HTML, CSS, and JavaScript, while implementing the server-side functionality with Python and Flask [23].

The AI probability panel displays the probability score for each retinal disease (acute or chronic CSC). These scores are generated from the last fully connected layer of the proposed deep learning model using the softmax activation function and allow users to measure the confidence of the AI model with its decision. The probabilities are illustrated with progress bars to enable users to intuitively perceive the model’s confidence.

The evidence heatmap panel reveals important regions in the OCT image while the model classifies the target label (eg, acute or chronic CSC). Gradient-weighted class activation mapping was adopted to highlight the important regions [24]. The activated regions were calculated using the feature-map gradients of the convolutional neural network (CNN) layer. The heatmap highlights the area of the image wherein the proposed model was used for classification. Moreover, users can zoom in or zoom out of the OCT images in the panel to observe the details of the pathologic regions.

The status panel displays the patient information of the current sample. Patient information included identification number, sex, and age. Users were able to identify the demographic information of a patient while analyzing a given image.

#### AI-CAD System CSC-Subtype Detection Model

To automatically classify a given OCT image into 2 different CSC subtypes, we use CNN-based architecture, VGG-16 [25]. The convolutional filters in CNN layers learn local patterns such as edges and textures, which is crucial for image recognition. Although other well-known CNN architectures, including VGG-19 and Resnet-50 [26], have been used previously, VGG-16 was selected in this study as it outperforms the others in our validation set. The proposed model uses spectral domain OCT (SD-OCT) images as input and predicts 1 of the 2 subtypes, that is, acute or chronic CSC. The detailed architecture of the proposed model is illustrated in Multimedia Appendix 2.

To train and evaluate the AI model, the data were randomly split into the training, validation, and test sets in an 8:1:1 ratio. The validation set was exclusively used to tune the hyperparameters of the model, and the test set was singularly used to evaluate the final performance of the model. We trained the proposed model using batch sizes of 64 and 30 epochs and Adam optimization [27] (learning rate: 0.0002). Moreover, we leverage transfer learning method to avoid overfitting. The details of transfer learning, data set construction, including collection, labeling, and preprocessing, are described in Multimedia Appendix 3.

### Observer Performance Test

**Figure 2:**
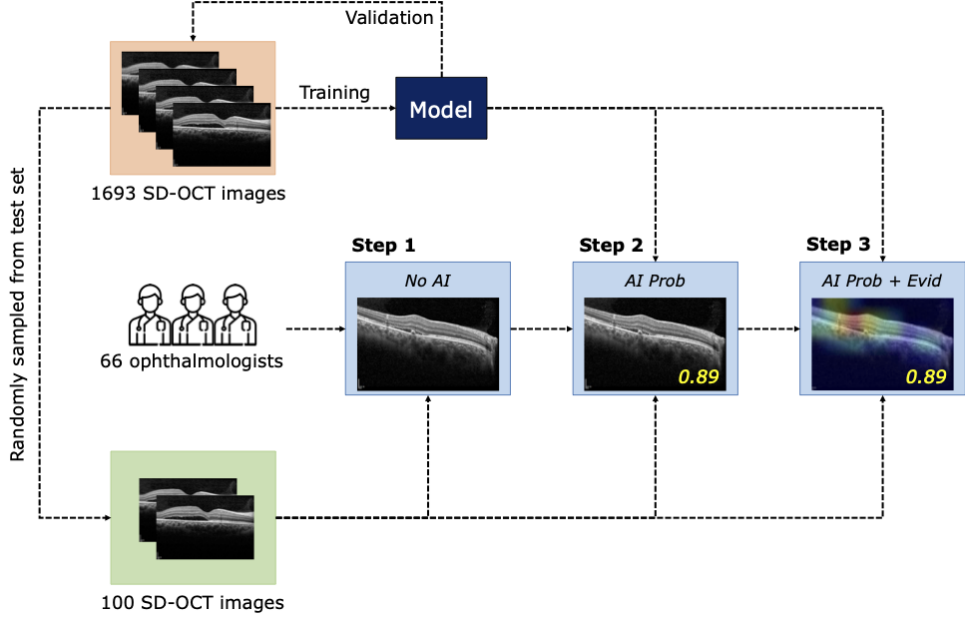
An illustration of the experimental procedure. In total, 66 ophthalmologists participated in the experiment. All observers were instructed to select one of the possible retinal diseases after reviewing the given SD-OCT image. In step 1, only the SD-OCT image was provided without any AI advice (ie, No AI). In step 2, the decision probability provided by AI was displayed on the system (ie, AI Prob). Finally, in step 3, the visual evidence of the AI decision was provided in addition to the AI probability panel (ie, AI Prob+Evid). The AI model was trained, validated, and tested using 1389, 141, and 163 images, respectively. The experiment was conducted using 100 images randomly sampled from the test set. AI: artificial intelligence; AI Prob+Evid: artificial intelligence assistance with a probability score and visual evidence heatmap; AI Prob: artificial intelligence assistance with a probability score; No AI: without artificial intelligence assistance; SD-OCT: spectral domain optical coherence tomography.

To investigate whether each component of the proposed system can assist in improving the diagnostic performance of ophthalmologists, a web-based experiment was conducted in which each participant was instructed to classify CSC subtypes from a given SD-OCT image. The experimental procedure comprised 3 steps (Figure 2). In the first step, observers had to identify the possible CSC subtype based on the SD-OCT image. The observers diagnosed retinal disease without artificial intelligence assistance (ie, *No AI*). In the subsequent step, an AI probability panel was provided to the observers (ie, artificial intelligence assistance with a probability score [*AI Prob*]). The AI probability panel shows the probability score of each retinal disease (acute or chronic). At the end of the step, both the AI probability panel and AI evidence heatmap panel were added to the system to provide a visual explanation to the observers (ie, artificial intelligence assistance with a probability score and visual evidence heatmap [*AI Prob+Evid*]).

In each step, all observers had to determine whether the given OCT image reflected acute or chronic CSC by selecting a button on the web system. The same OCT image was used in the 3 steps. As 100 SD-OCT images were used in our experiment, each participant assessed 300 cases (ie, 3 steps × 100 images) in total. The 100 images were randomly extracted from the test set that was not used to train our model. The step-by-step user interface for the observer performance test is illustrated in Multimedia Appendix 4.

The study recruited 66 participants, including 36 retina and 30 nonretina specialists. The retina specialists were medical doctors who had completed 1-2 years of the retina fellowship training program. In contrast, nonretina specialists were board-certified ophthalmologists who were not specialized in the retina. The detailed information of the 66 participants is summarized Multimedia Appendix 5.

### Statistical Analysis

Receiver operating characteristic analysis was conducted to evaluate the performance of the proposed model and ophthalmologists in classifying the CSC subtypes. Thereafter, the receiver operating characteristic curve with the true-positive and false-positive rates was plotted to measure the area under the receiver operating characteristic curve (AUROC) score. Friedman [28] test, followed by the Wilcoxon signed-rank test, was used to quantify the differences among the 3 different conditions (ie, *No AI*, *AI Prob*, and *AI Prob+Evid*) [29]. A 2-tailed *t* test was used to compare diagnostic performance between the nonretina and retina specialists.

## Results

### CSC-Subtype Detection Model Performance

The proposed model exhibited high accuracy, sensitivity, and specificity values of 96.3%, 97.1%, and 95.7%, respectively (n=163). The model achieved 98.4% (n=163) of the AUROC which outperforms Resnet-50 (n=163; AUROC 87.9%) and VGG-19 (n=163; AUROC 96.1%). The model failed to accurately predict 6 cases only in our test set. Importantly, the training AUROC of 95.6% (n=163) indicates that our model strikes a balance between avoiding overfitting and underfitting, further affirming its reliability.

### Performance Comparison Between the AI-CAD System and Ophthalmologists

The diagnostic performance of the AI-CAD system was compared with that of ophthalmologists. The AUROCs were calculated to evaluate the AI-CAD and human predictive abilities for 100 images randomly extracted from the test set. The AI-CAD system (AUROC 99.5%; n=100) outperformed both retina (AUROC 92.1%; n-36) and nonretina (AUROC 87.8%; n=30) specialists.

### Diagnostic Performance of Ophthalmologists

The retinal-disease detection performance of the 36 retina and 30 nonretina specialists were evaluated under 3 different conditions (ie, *No AI*, *AI Prob,* and *AI Prob+Evid*; Table 1). The results of the Friedman test followed by the Wilcoxon signed-rank test revealed significant differences in diagnostic performance among the 3 different conditions for retina (statistic=59.5, *df*=2; *P*<.001) and nonretina (statistic=44.4, *df*=2; *P*<.001) specialists. In particular, the retina specialists who were provided with the AI probability panel and AI evidence heatmap panel (ie, *AI Prob+Evid*) achieved the highest mean diagnostic performance (AUROC 95.8%, 95% CI 0.948-0.969; n=36) compared with those subjected to other conditions (*No AI*: 0.921, 95% CI 0.907-0.935; *P*<.001; and *AI Prob*: 0.956, 95% CI 0.946-0.967; *P*<.05). The nonretina specialists also displayed their best performance (0.929, 95% CI 0.913-0.946) with numerical and visual information compared with when they were subjected to other conditions (*No AI*: 0.878, 95% CI 0.860-0.895; *P*<.001; and *AI Prob*: 0.922, 95% CI 0.905-0.940; *P*<.001).

**Table 1:** Diagnostic performance of retina and nonretina specialists.

Step and observer group			AUROC^a^ (95% CI)		Sensitivity (95% CI)		Specificity (95% CI)		Accuracy (95% CI)	
**Step 1: *No AI*^b^**										
	Nonretina specialist			0.878 (0.860-0.895)		0.934 (0.910-0.958)		0.821 (0.794-0.848)		0.859 (0.840-0.878)
	Retina specialist			0.921 (0.907-0.935)		0.930 (0.905-0.954)		0.912 (0.890-0.933)		0.918 (0.904-0.932)
	Both			0.901 (0.889-0.913)		0.932 (0.915-0.949)		0.870 (0.850-0.890)		0.891 (0.878-0.905)
**Step 2: *AI Prob*^c^**										
		Nonretina specialist		0.922 (0.905-0.940)		0.977 (0.966-0.989)		0.867 (0.837-0.897)		0.905 (0.884-0.926)
		Retina specialist		0.956 (0.946-0.966)		0.969 (0.954-0.984)		0.943 (0.924-0.962)		0.952 (0.940-0.964)
		Both		0.941 (0.930-0.951)		0.973 (0.963-0.982)		0.909 (0.889-0.928)		0.930 (0.918-0.943)
**Step 3: *AI Prob+Evid*^d^**										
	Nonretina specialist			0.929 (0.913-0.946)		0.982 (0.974-0.990)		0.876 (0.846-0.906)		0.912 (0.892-0.933)
	Retina specialist			0.958 (0.948-0.969)		0.971 (0.956-0.987)		0.945 (0.926-0.964)		0.954 (0.942-0.966)
	Both			0.945 (0.935-0.955)		0.976 (0.967-0.985)		0.914 (0.895-0.933)		0.935 (0.923-0.947)

^a^AUROC: area under the receiver operating characteristic curve.

^b^*No AI*: without artificial intelligence assistance.

^c^*AI Prob*: artificial intelligence assistance with a probability score.

^d^*AI Prob+Evid*: artificial intelligence assistance with a probability score and visual evidence heatmap.

### Benefits of the AI-CAD System in CSC-Subtype Classification

**Figure 3:**
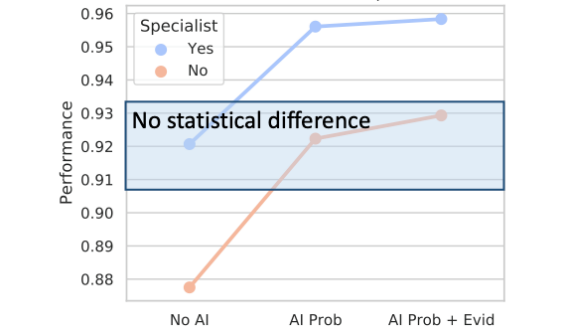
Comparison of the diagnostic performance of retina and nonretina specialists. The *t* test results revealed no significant difference in AUROC between nonretina specialists supported by AI-CAD (AI Prob: 0.922, 95% CI 0.905 to 0.940; *P*=.88 or AI Prob+Evid: 0.929, 95% CI 0.913 to 0.946; *P*=.42) and retina specialists with no AI support (No AI: 0.921, 95% CI 0.907 to 0.935). This implies that nonretina specialists can achieve expert-level performance with the proposed AI-CAD system. AI: artificial intelligence; AI Prob+Evid: artificial intelligence assistance with a probability score and visual evidence heatmap; AI Prob: artificial intelligence assistance with a probability score; AI-CAD: artificial intelligence–based computer-aided diagnosis; AUROC: area under the receiver operating characteristic curve; No AI: without artificial intelligence assistance.

This study investigated how the AI-CAD system can help nonretina specialists detect retinal diseases. The *t* test results revealed no significant differences in AUROC between the nonretina specialists supported by the AI-CAD system (*AI Prob*: 0.922, 95% CI 0.905-0.940; *P*=.88; and *AI Prob+Evid*: 0.929, 95% CI 0.913-0.946; *P*=.42) and retina specialists not supported by the AI-CAD system (*No AI*: 0.921, 95% CI 0.907-0.935). This finding demonstrates that nonretina specialists can achieve expert-level diagnostic performance with the support of the proposed AI CAD system (Figure 3).

### Changes in Clinical Diagnosis With the Support of the AI-CAD System

To evaluate the proposed system’s positive effect in assisting ophthalmologists, the number of positive (ie, false negative to true positive and false positive to true negative) and negative (ie, true positive to false negative and true negative to false positive) changes that could be observed between the *No AI* and *AI Prob+Evid* conditions were recorded. Chronic CSC was set as the positive class. Overall, 42.8% (307/718) of the misclassified cases under the *No AI* condition were accurately classified in the *AI Prob+Evid* condition (Table 2). In particular, 106 false-negative cases turned into true positives after using the proposed AI-CAD system, implying that the proposed system is useful for ophthalmologists in distinguishing between acute and chronic CSC.

**Table 2:** Clinical diagnosis changes after AI-CAD^a^ use.

Observer group	Clinical diagnosis								
	Positively changed by AI^b^			Sum of positive changes		Negatively changed by AI			Sum of negative changes
	FN^c^ to TP^d^	FP^e^ to TN^f^			TP to FN		TN to FP		
Nonretina specialist, n	52	115	167		3		5	8	
Retina specialist, n	54	86	140		3		6	9	
All, n	106	201	307		6		11	17	

^a^AI-CAD: artificial intelligence–based computer-aided diagnosis system.

^b^AI: artificial intelligence.

^c^FN: false negative.

^d^TP: true positive.

^e^FP: false positive.

^f^TN: true negative.

## Discussion

### Principal Findings

This study proposed the development of an AI-CAD system to assist ophthalmologists in distinguishing chronic from acute CSC. In particular, the proposed system provides (1) the probability of retinal disease and (2) visual evidence to effectively assist ophthalmologists in their clinical decisions. To evaluate the effectiveness of the proposed AI-CAD system in enhancing ophthalmologists’ clinical decision-making, a within-subject user study involving 66 ophthalmologists was conducted. The extensive experiments demonstrated that the proposed AI-CAD system effectively assists ophthalmologists in improving their diagnostic performance for retinal disease.

The proposed deep neural network in the AI-CAD system achieved a high retinal-disease detection performance of 99.5% (n=100) of the AUROC, outperforming all 66 ophthalmologists who participated in the experiment. The high performance of the proposed AI-CAD model implies that it can lessen the heavy workloads and reduce potential errors by clinicians [8,9]. The quality of clinical services can be improved by using a deep learning model that guarantees consistent and high-level detection performance for retinal disease.

The observer performance test revealed that the proposed AI-CAD system can effectively help ophthalmologists diagnose retinal disease. The experimental results revealed that the diagnostic performance of the retina specialists and nonretina specialists increased by 3.5% (n=36) and 4.4% (n=30), respectively (Table 1). This signifies that the proposed system successfully improved the ability of ophthalmologists to detect retinal disease. In particular, the performance difference between cases with and without the AI-CAD system was higher in the nonretina specialist group (n=30; 4.4%) than that in the retina specialist group (n=36; 3.5%), implying that the nonretina specialist group tended to receive support more than the retina specialist group. This finding is consistent with that of a prior study, which revealed that relatively less-skilled physicians exhibited greater improvement in the detection of pulmonary disease compared with that of skilled physicians [6,7]. Moreover, ophthalmologists achieved an even higher AUROC (retina specialists: 0.958; nonretina specialists: 0.929) if AI diagnosis information was availed with its visual explanation (gradient-weighted class activation mapping). This indicates that providing probability scores with a visual explanation is more useful for ophthalmologists than simply displaying the probability scores alone, thereby exhibiting consistency with prior work that revealed the usefulness of visual modality in detecting diseases [7].

### Practical Issues

Deploying a clinical decision support system (CDSS) in real-world health care settings presents a set of practical challenges. Chief among these concerns is the system’s susceptibility to errors, which can undermine trust in AI-driven solutions. To address this, a dynamic training model becomes indispensable. In the realm of academia, researchers have embraced the “Human-In-the-Loop” paradigm to tackle this issue [30-32]. This approach involves the seamless integration of human oversight and intervention into the CDSS’s decision-making processes. By empowering human experts to review and amend the system's outputs, we expedite the identification and rectification of errors. Consequently, this iterative feedback mechanism bolsters the CDSS's trustworthiness and reliability in real-world applications, bringing it into closer alignment with user expectations and requirements.

Another pivotal concern pertains to the system's security. Safeguarding patient data and upholding the integrity of the CDSS is paramount within the health care domain. This demands the implementation of robust encryption, stringent access controls, and regular security audits to protect sensitive information and prevent unauthorized access or data breaches. Furthermore, continuous monitoring and timely updates to the CDSS are essential for addressing emerging security threats and vulnerabilities, ensuring a high level of security in real-world applications.

By concurrently addressing error mitigation and security, CDSS developers and health care professionals can collaborate in creating a more dependable and trustworthy system that serves the best interests of both patients and medical practitioners.

### Limitations

This study has some limitations. First, all images were acquired from a single OCT device located at a single academic center. Although the data set was sufficient to train and validate the proposed model for distinguishing between CSC subtypes, external validation with a different center is needed. Second, experiments were conducted using the web-based AI-CAD system developed in this study. Thus, the environment was relatively different from that of actual clinical practice. However, we attempted to design and develop a user-friendly AI-CAD system under the supervision of retina specialists. Third, considering that the model's training data comprises images taken exclusively by the Heidelberg Spectralis device (Heidelberg Engineering Inc), its performance might be insufficient when dealing with images from different devices. Future studies should prioritize (1) extending the proposed AI-CAD system to other retinal diseases, such as AMD and diabetic retinopathy; (2) developing strategies to improve the reliability of doctors when using the AI-CAD system; and (3) exploring the application of transfer learning techniques to address the challenges arising from variations in devices.

### Comparison With Prior Work

This study has several implications. First, to the best of our knowledge, this study is the first attempt to develop and evaluate an AI-CAD system for the detection of retinal disease using OCT. Prior studies have developed AI-CAD systems for the detection of pulmonary disease and evaluated their effectiveness [6,33,34]. However, minimal attention has been focused on the application of an AI-CAD system for the diagnosis of retinal diseases, such as CSC and AMD. In this study, an AI-CAD system that can assist ophthalmologists in identifying retinal diseases was developed and its usefulness in detecting retinal disease was evaluated.

Second, the proposed AI-CAD system is potentially useful for small or local medical care centers where retina specialists are unavailable. Unlike in large-scale medical care centers or hospitals, retina specialists are rarely found in small or local centers. Diagnosing subtypes of retinal diseases (ie, acute vs chronic CSC [8] and polypoidal choroidal vasculopathy vs retinal angiomatous proliferation [10]) requires more elaborate expertise than simply screening abnormal cases (ie, normal vs CSC [8]), and the proposed AI-CAD system exhibits higher performance than that of retina specialists with over 10 years of experience, implying that the proposed system potentially plays an important role in such cases. In CSC, assessing the chronicity of the disease at the time of diagnosis is crucial for selecting an appropriate course of treatment or forecasting its prognosis [8,15,22]. Acute CSC typically follows a self-limiting natural course, whereas chronic CSC with or without sustained SRF may be associated with irreversible vision loss or may require active intervention, such as intravitreal antivascular endothelial growth factor injections or photodynamic therapy, all of which are intended to prevent long-term visual loss that can lower the patient’s quality of life. Further, on comparing diagnostic performance between nonretina and retina specialists, the experimental results of this study demonstrate that nonretina specialists can achieve retina specialist-level performance with the support of the proposed AI-CAD system (Figure 3). This implies that the proposed system can alleviate the heavy workload of ophthalmologists who have expert-level diagnostic performance and facilitate the decision-making process of less-skilled ophthalmologists (nonretina specialists) by improving their diagnostic performance.

### Conclusions

To the best of our knowledge, this study is the first attempt to design, develop, and evaluate an AI-CAD system for the detection of retinal disease using OCT. First, an AI-CAD system was developed with a high-performance deep learning model. Thereafter, an observer performance test was conducted with the proposed system to determine the ability of the system to assist ophthalmologists in diagnosing retinal diseases. The results indicated that the proposed AI-CAD system can provide retinal expert-level diagnostic performance and help ophthalmologists improve their diagnostic performance in detecting CSC subtypes. Thus, the proposed AI-CAD system can alleviate the heavy workload of ophthalmologists and help in the decision-making process involved in detecting CSC subtypes. As a base study, this study demonstrates the usefulness and effectiveness of using an AI-CAD system in detecting retinal diseases, particularly CSC subtypes. In the future, the proposed AI-CAD system may be easily extended to the detection of other retinal diseases, such as AMD, diabetic retinopathy, and branch retinal vein occlusion.

## Appendix

Multimedia Appendix 1 Representative cases of acute and chronic central serous chorioretinopathy.

Multimedia Appendix 2 An illustration of the proposed deep learning model based on VGG-16 architecture.

Multimedia Appendix 3 Method details.

Multimedia Appendix 4 A step-by-step illustration of the observer performance test.

Multimedia Appendix 5 Information and test results of the ophthalmologists who participated in the observer performance test.

## Abbreviations

AI-CAD: artificial intelligence–based computer-aided diagnosis
AI: artificial intelligence
AI Prob+Evid: artificial intelligence assistance with a probability score and visual evidence heatmap
AI Prob: artificial intelligence assistance with a probability score
AMD: age-related macular degeneration
AUROC: area under the receiver operating characteristic curve
CAD: computer-aided diagnosis
CDSS: clinical decision support system
CNN: convolutional neural network
CSC: central serous chorioretinopathy
FA: fluorescein angiography
ICGA: indocyanine green angiography
No AI: without artificial intelligence assistance
OCT: optical coherence tomography
RPE: retinal pigment epithelium
SD-OCT: spectral domain optical coherence tomography
SRF: subretinal fluid

## Data Availability

The data are not available for public access because of patient privacy concerns but are available from the corresponding author upon reasonable request.

## References

[1] Doi K (2007). Computer-aided diagnosis in medical imaging: historical review, current status and future potential. Comput Med Imaging Graph.

[2] Chan HP, Samala RK, Hadjiiski LM (2020). CAD and AI for breast cancer-recent development and challenges. Br J Radiol.

[3] Brown MS, Lo P, Goldin JG, Barnoy E, Kim GHJ, McNitt-Gray MF, Aberle DR (2014). Toward clinically usable CAD for lung cancer screening with computed tomography. Eur Radiol.

[4] Mittal A, Kaur M (2016). Computer-aided-diagnosis in colorectal cancer: a survey of state of the art techniques.

[5] Martínez-Murcia FJ, Górriz JM, Ramírez J, Puntonet CG, Salas-González D (2012). Computer aided diagnosis tool for Alzheimer's disease based on Mann-Whitney-Wilcoxon U-Test. Expert Syst Appl.

[6] Hwang EJ, Park S, Jin KN, Kim JI, Choi SY, Lee JH, Goo JM, Aum J, Yim JJ, Cohen JG, Ferretti GR, Park CM (2019). Development and validation of a deep learning-based automated detection algorithm for major thoracic diseases on chest radiographs. JAMA Netw Open.

[7] Choi SY, Park S, Kim M, Park J, Choi YR, Jin KN (2021). Evaluation of a deep learning-based computer-aided detection algorithm on chest radiographs: case-control study. Medicine (Baltimore).

[8] Yoon J, Han J, Park JI, Hwang JS, Han JM, Sohn J, Park KH, Hwang DD (2020). Optical coherence tomography-based deep-learning model for detecting central serous chorioretinopathy. Sci Rep.

[9] Yoon J, Han J, Ko J, Choi S, Park JI, Hwang JS, Han JM, Jang K, Sohn J, Park KH, Hwang DDJ (2022). Classifying central serous chorioretinopathy subtypes with a deep neural network using optical coherence tomography images: a cross-sectional study. Sci Rep.

[10] Hwang DDJ, Choi S, Ko J, Yoon J, Park JI, Hwang JS, Han JM, Lee HJ, Sohn J, Park KH, Han J (2021). Distinguishing retinal angiomatous proliferation from polypoidal choroidal vasculopathy with a deep neural network based on optical coherence tomography. Sci Rep.

[11] Esteva A, Kuprel B, Novoa RA, Ko J, Swetter SM, Blau HM, Thrun S (2017). Dermatologist-level classification of skin cancer with deep neural networks. Nature.

[12] Wang M, Munch IC, Hasler PW, Prünte C, Larsen M (2008). Central serous chorioretinopathy. Acta Ophthalmol.

[13] Breukink MB, Dingemans AJ, den Hollander AI, Keunen JE, MacLaren RE, Fauser S, Querques G, Hoyng CB, Downes SM, Boon CJ (2017). Chronic central serous chorioretinopathy: long-term follow-up and vision-related quality of life. Clin Ophthalmol.

[14] Sahin A, Bez Y, Kaya MC, Türkcü FM, Sahin M, Yüksel H (2014). Psychological distress and poor quality of life in patients with central serous chorioretinopathy. Semin Ophthalmol.

[15] van Rijssen TJ, van Dijk EHC, Yzer S, Ohno-Matsui K, Keunen JEE, Schlingemann RO, Sivaprasad S, Querques G, Downes SM, Fauser S, Hoyng CB, Piccolino FC, Chhablani JK, Lai TYY, Lotery AJ, Larsen M, Holz FG, Freund KB, Yannuzzi LA, Boon CJF (2019). Central serous chorioretinopathy: towards an evidence-based treatment guideline. Prog Retin Eye Res.

[16] Shinojima A, Hirose T, Mori R, Kawamura A, Yuzawa M (2010). Morphologic findings in acute central serous chorioretinopathy using spectral domain-optical coherence tomography with simultaneous angiography. Retina.

[17] Song IS, Shin YU, Lee BR (2012). Time-periodic characteristics in the morphology of idiopathic central serous chorioretinopathy evaluated by volume scan using spectral-domain optical coherence tomography. Am J Ophthalmol.

[18] Kaye R, Chandra S, Sheth J, Boon CJF, Sivaprasad S, Lotery A (2020). Central serous chorioretinopathy: an update on risk factors, pathophysiology and imaging modalities. Prog Retin Eye Res.

[19] Ko J, Han J, Yoon J, Park JI, Hwang JS, Han JM, Park KH, Hwang DDJ (2022). Assessing central serous chorioretinopathy with deep learning and multiple optical coherence tomography images. Sci Rep.

[20] Montero JA, Ruiz-Moreno JM (2005). Optical coherence tomography characterisation of idiopathic central serous chorioretinopathy. Br J Ophthalmol.

[21] Spaide RF, Campeas L, Haas A, Yannuzzi LA, Fisher YL, Guyer DR, Slakter JS, Sorenson JA, Orlock DA (1996). Central serous chorioretinopathy in younger and older adults. Ophthalmology.

[22] Daruich A, Matet A, Dirani A, Bousquet E, Zhao M, Farman N, Jaisser F, Behar-Cohen F (2015). Central serous chorioretinopathy: recent findings and new physiopathology hypothesis. Prog Retin Eye Res.

[23] Grinberg M (2018). Flask Web Development: Developing Web Applications with Python.

[24] Selvaraju RR, Cogswell M, Das A, Vedantam R, Parikh D, Batra D (2017). Grad-CAM: visual explanations from deep networks via gradient-based localization.

[25] Simonyan K, Zisserman A (2015). Very deep convolutional networks for large-scale image recognition. https://arxiv.org/abs/1409.1556.

[26] He K, Zhang X, Ren S, Sun J (2016). Deep residual learning for image recognition.

[27] Kingma DP, Ba J (2015). Adam: a method for stochastic optimization. Proceedings of the 3rd International Conference on Learning Representations.

[28] Friedman M (1937). The use of ranks to avoid the assumption of normality implicit in the analysis of variance. J Am Stat Assoc.

[29] Wilcoxon F, Kotz S, Johnson NL (1992). Individual comparisons by ranking methods. Springer Series in Statistics: Breakthroughs in Statistics.

[30] Wu X, Xiao L, Sun Y, Zhang J, Ma T, He L (2022). A survey of human-in-the-loop for machine learning. Future Gener Comput Syst.

[31] Sorantin E, Grasser MG, Hemmelmayr A, Tschauner S, Hrzic F, Weiss V, Lacekova J, Holzinger A (2022). The augmented radiologist: artificial intelligence in the practice of radiology. Pediatr Radiol.

[32] Le EPV, Wang Y, Huang Y, Hickman S, Gilbert FJ (2019). Artificial intelligence in breast imaging. Clin Radiol.

[33] Nam JG, Park S, Hwang EJ, Lee JH, Jin KN, Lim KY, Vu TH, Sohn JH, Hwang S, Goo JM, Park CM (2019). Development and validation of deep learning-based automatic detection algorithm for malignant pulmonary nodules on chest radiographs. Radiology.

[34] Sung J, Park S, Lee SM, Bae W, Park B, Jung E, Seo JB, Jung KH (2021). Added value of deep learning-based detection system for multiple major findings on chest radiographs: a randomized crossover study. Radiology.

