# Supplementary material for: Developing and Evaluating an AI-Based Computer-Aided Diagnosis System for Retinal Disease: Diagnostic Study for Central Serous Chorioretinopathy
Source: J Med Internet Res. 2023 Nov 29;25:e48142. doi: 10.2196/48142 (PMC10719821; doi:10.2196/48142)
Supplement: Multimedia Appendix 1 [file jmir_v25i1e48142_app1.docx]

**Multimedia Appendix 1.** Representative cases of acute and chronic CSC.

In acute CSC (A), IR exhibited SRF-induced decreased reflectance at the foveal center. FA revealed leakage of dye in the ink blot pattern, and OCT showed increased subfoveal choroidal thickness and the presence of SRF. In chronic CSC (B), AF demonstrated the gravitational track of RPE atrophy, and OCT revealed RPE irregularity and a disrupted outer retinal layer with atrophic changes of the photoreceptor layer. AF, fundus autofluorescence; CSC, central serous chorioretinopathy; FA, fluorescein angiography; FP, color fundus photography; IR, infrared; ICGA, indocyanine green angiography; OCT, optical coherence tomography

**
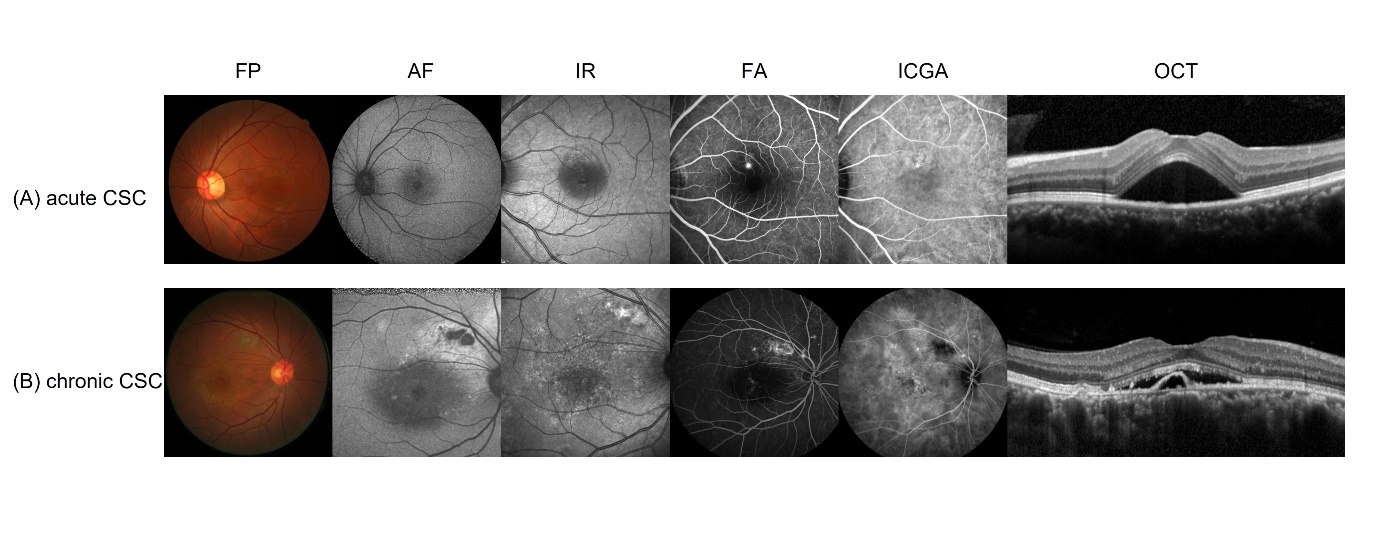
**
