# Supplementary material for: Developing and Evaluating an AI-Based Computer-Aided Diagnosis System for Retinal Disease: Diagnostic Study for Central Serous Chorioretinopathy
Source: J Med Internet Res. 2023 Nov 29;25:e48142. doi: 10.2196/48142 (PMC10719821; doi:10.2196/48142)
Supplement: Multimedia Appendix 2 [file jmir_v25i1e48142_app2.docx]

**Multimedia Appendix 2.** An illustration of the proposed deep learning model based on VGG-16 architecture.

The proposed model comprises 13 CNN layers, followed by a rectified linear unit (ReLU) activation function, four max-pooling layers, one global average pooling layer, and two linear layers with dropouts and softmax activation. The dropout potentially helped our model avoid overfitting, and a fully connected layer was a traditional multilayered perceptron^4^. The final output layer with a softmax activation function was used to predict the CSC subtype: acute or chronic CSC.

**Figure S1.** An illustration of the proposed deep learning model based on VGG-16 architecture.

**
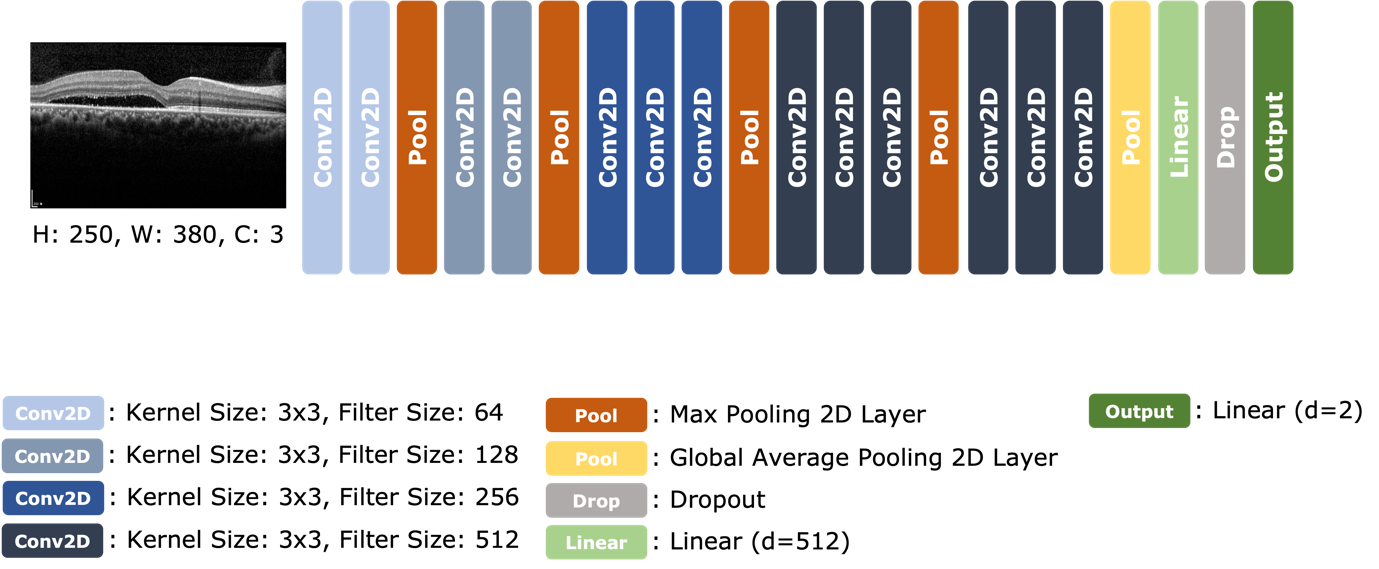
**
