# Supplementary material for: Developing and Evaluating an AI-Based Computer-Aided Diagnosis System for Retinal Disease: Diagnostic Study for Central Serous Chorioretinopathy
Source: J Med Internet Res. 2023 Nov 29;25:e48142. doi: 10.2196/48142 (PMC10719821; doi:10.2196/48142)
Supplement: Multimedia Appendix 3 [file jmir_v25i1e48142_app3.docx]

**Multimedia Appendix 3.** Method details.

**Transfer Learning**

We applied the transfer learning method to avoid overfitting, thus, training our model faster. In particular, we first initialized 13 CNN layers using pretrained weights that were part of VGG-16. Note that the pretrained weights were acquired from the large-scale image dataset ImageNet. We subsequently froze the CNN layers during training. The total number of trainable parameters of the proposed deep neural network was 263,682 out of the 14,978,370 model parameters.

**Dataset Collection and Labeling**

We analyzed the records of the patients who visited Hangil Eye Hospital between June 2017 and June 2021. We used spectral domain optical coherence tomography (SD-OCT) (Heidelberg Spectralis, Heidelberg Engineering, Heidelberg, Germany) images of patients with CSC. All CSC cases were diagnosed using fundus examination, ﬂuorescein angiography (FA), indocyanine green angiography (ICGA), and OCT images by independent retinal specialists (DDH and JSH). A confocal scanning laser ophthalmoscope (Heidelberg Retina Angiograph, HRA; Heidelberg Engineering, Heidelberg, Germany) was used to simultaneously perform FA and ICGA in all CSC cases. One eye per patient was selected for this study based on one visit per patient. Based on the retinal pigment epithelium (RPE) or photoreceptor status and symptom duration, patients were classified into acute and chronic CSC groups: (1) acute CSC with subretinal fluid (SRF) lasting ≤ 4 months and (2) chronic atrophic CSC with definite RPE and photoreceptor atrophy with or without SRF^3^ (see **Multimedia Appendix 2**). In cases of disagreement, a third retinal specialist (JMH) evaluated the discrepancy and discussed the case with other specialists. After discussion, all discrepancies were resolved by consensus. Our analysis excluded data that indicated the presence of other potentially conflicting retinal pathologies, such as age-related macular degeneration, polypoidal choroidal vasculopathy, pachychoroid neovasculopathy, and pachychoroid pigment epitheliopathy.

**Data Preprocessing and Augmentation**

To use the obtained 1,693 SD-OCT images as inputs for a deep neural network, we initially cropped a given 596 x 1,264-sized SD-OCT image into a 500 × 764 RGB image. We then downsampled a cropped 500 × 764 image into a 250 × 384 RGB image to feed the deep neural network, which only takes fixed-size images as inputs. Data augmentation was performed to avoid overfitting, that is, we (i) flipped images horizontally, (ii) shifted the width/height of images randomly in the range of -1.0–1.0 pixels, and (iii) randomly rotated images up to 15° to construct a robust model based on a variety of input images. Note that the data augmentation process was performed only during the training phase.
