# Supplementary material for: Developing and Evaluating an AI-Based Computer-Aided Diagnosis System for Retinal Disease: Diagnostic Study for Central Serous Chorioretinopathy
Source: J Med Internet Res. 2023 Nov 29;25:e48142. doi: 10.2196/48142 (PMC10719821; doi:10.2196/48142)
Supplement: Multimedia Appendix 4 [file jmir_v25i1e48142_app4.docx]

**Multimedia Appendix 4.** A step-by-step illustration of the observer performance test.

This figure illustrates the step-by-step user interface of the proposed AI-CAD system used in the observer performance test. In step 1, observers were asked to select one of the possible retinal diseases (i.e., acute or chronic CSC) from the SD-OCT image. Here, the observers diagnosed retinal disease without any AI support. Upon completing disease identification, observers proceeded to Step 2. In Step 2, the AI decision score was availed to the observers. The AI Probability Panel shows the probability scores for each retinal disease (acute or chronic). These scores were generated from the last fully connected layer of the proposed deep learning model with a softmax activation function and allowed users to determine the AI model’s confidence with its decision. Note that the probabilities are illustrated with progress bars to enable users to intuitively perceive the confidence of the model. Finally, in Step 3, the AI Evidence Heatmap Panel was provided. Visual evidence represents the important regions in the OCT image when our proposed model classifies the target label (e.g., acute or chronic). To highlight important regions, we adopted gradient-weighted class activation mapping (Grad-CAM). The activated regions were calculated using the feature-map gradients of the CNN layer. The heatmap highlights the area of the image in which the proposed model was used for classification. Note that observers could scroll to zoom OCT images in or out to observe the details of the pathologic regions.

**
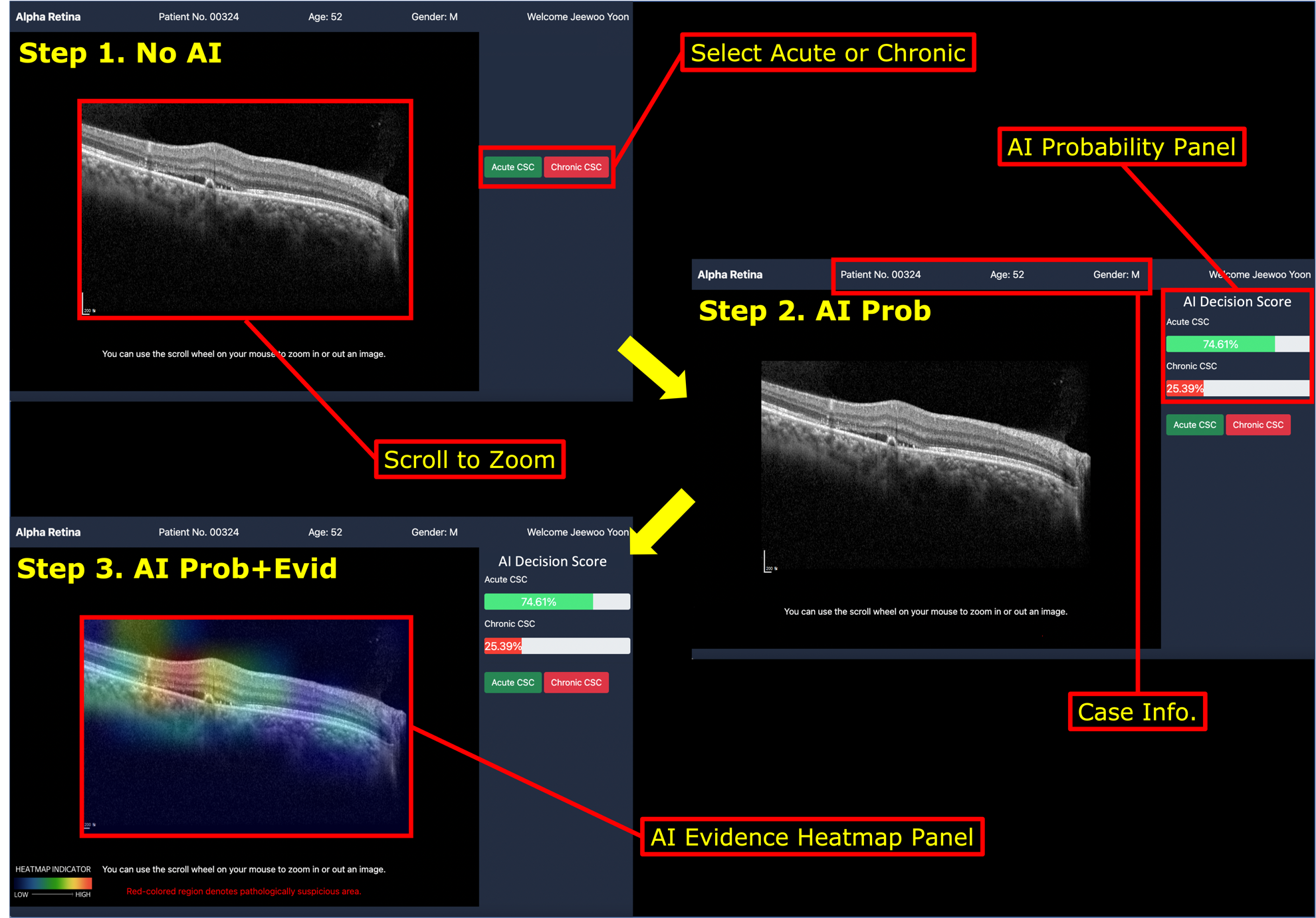
**
