# Supplementary material for: Developing and Evaluating an AI-Based Computer-Aided Diagnosis System for Retinal Disease: Diagnostic Study for Central Serous Chorioretinopathy
Source: J Med Internet Res. 2023 Nov 29;25:e48142. doi: 10.2196/48142 (PMC10719821; doi:10.2196/48142)
Supplement: Multimedia Appendix 5 [file jmir_v25i1e48142_app5.docx]

**Multimedia Appendix 5.** Information and test results of the ophthalmologists who participated in the observer performance test.

| **Name** | **Oph. (yrs)** | **Ret. (yrs)** | ***No AI*** | ***AI Prob*** | ***AI Prob+Evid*** |
| --- | --- | --- | --- | --- | --- |
| RS1 | 15 | 12 | 0.88 | 0.92 | 0.93 |
| RS2 | 16 | 9 | 0.87 | 0.9 | 0.9 |
| RS3 | 14 | 11 | 0.88 | 0.96 | 0.96 |
| RS4 | 7 | 4 | 0.98 | 0.99 | 0.99 |
| RS5 | 8 | 8 | 0.9 | 0.97 | 0.97 |
| RS6 | 5 | 5 | 0.86 | 0.95 | 0.97 |
| RS7 | 7 | 4 | 0.92 | 0.97 | 0.98 |
| RS8 | 3 | 3 | 0.93 | 0.94 | 0.94 |
| RS9 | 13 | 10 | 0.84 | 0.9 | 0.91 |
| RS10 | 9 | 9 | 0.94 | 0.97 | 0.97 |
| RS11 | 5 | 2 | 0.93 | 0.95 | 0.96 |
| RS12 | 7 | 7 | 0.95 | 0.99 | 0.99 |
| RS13 | 10 | 7 | 0.93 | 0.98 | 0.98 |
| RS14 | 25 | 22 | 0.87 | 1 | 0.99 |
| RS15 | 10 | 10 | 0.93 | 0.97 | 0.97 |
| RS16 | 11 | 11 | 0.93 | 0.99 | 0.99 |
| RS17 | 14 | 11 | 0.97 | 0.98 | 0.98 |
| RS18 | 8 | 8 | 0.97 | 0.98 | 0.98 |
| RS19 | 5 | 1 | 0.82 | 0.86 | 0.86 |
| RS20 | 14 | 11 | 0.97 | 0.99 | 0.99 |
| RS21 | 21 | 18 | 0.92 | 0.96 | 0.96 |
| RS22 | 22 | 19 | 0.97 | 0.98 | 0.98 |
| RS23 | 17 | 14 | 0.91 | 0.91 | 0.92 |
| RS24 | 5 | 1 | 0.86 | 0.92 | 0.93 |
| RS25 | 11 | 11 | 0.91 | 0.97 | 0.99 |
| RS26 | 11 | 11 | 0.91 | 0.92 | 0.92 |
| RS27 | 8 | 8 | 0.86 | 0.87 | 0.87 |
| RS28 | 12 | 12 | 0.94 | 0.97 | 0.97 |
| RS29 | 15 | 12 | 0.97 | 0.97 | 0.97 |
| RS30 | 17 | 14 | 0.89 | 0.91 | 0.91 |
| RS31 | 18 | 15 | 0.94 | 0.96 | 0.96 |
| RS32 | 15 | 12 | 0.9 | 0.9 | 0.9 |
| RS33 | 21 | 18 | 0.98 | 0.98 | 0.98 |
| RS34 | 6 | 3 | 0.91 | 0.94 | 0.93 |
| RS35 | 13 | 13 | 0.95 | 0.98 | 0.98 |
| RS36 | 8 | 8 | 0.95 | 0.97 | 0.97 |
| NRS1 | 21 | 0 | 0.89 | 0.93 | 0.93 |
| NRS2 | 20 | 0 | 0.86 | 0.92 | 0.92 |
| **Name** | **Oph. (yrs)** | **Ret. (yrs)** | ***No AI*** | ***AI Prob*** | ***AI Prob+Evid*** |
| NRS3 | 18 | 15 | 0.79 | 0.83 | 0.83 |
| NRS4 | 16 | 0 | 0.71 | 0.74 | 0.77 |
| NRS5 | 18 | 0 | 0.83 | 0.9 | 0.9 |
| NRS6 | 15 | 0 | 0.91 | 0.95 | 0.95 |
| NRS7 | 14 | 0 | 0.9 | 0.88 | 0.91 |
| NRS8 | 13 | 0 | 0.88 | 0.95 | 0.96 |
| NRS9 | 15 | 0 | 0.85 | 0.86 | 0.86 |
| NRS10 | 5 | 0 | 0.93 | 0.93 | 0.93 |
| NRS11 | 12 | 0 | 0.89 | 0.96 | 0.97 |
| NRS12 | 13 | 0 | 0.83 | 0.9 | 0.93 |
| NRS13 | 10 | 0 | 0.87 | 0.95 | 0.96 |
| NRS14 | 0 | 0 | 0.9 | 0.93 | 0.94 |
| NRS15 | 0 | 0 | 0.93 | 0.95 | 0.95 |
| NRS16 | 0 | 0 | 0.89 | 0.9 | 0.91 |
| NRS17 | 19 | 0 | 0.86 | 0.85 | 0.85 |
| NRS18 | 33 | 0 | 0.83 | 0.86 | 0.86 |
| NRS19 | 9 | 0 | 0.75 | 0.79 | 0.79 |
| NRS20 | 3 | 0 | 0.93 | 0.96 | 0.98 |
| NRS21 | 3 | 0 | 0.88 | 0.95 | 0.95 |
| NRS22 | 12 | 0 | 0.85 | 0.88 | 0.89 |
| NRS23 | 8 | 0 | 0.89 | 0.96 | 0.96 |
| NRS24 | 8 | 0 | 0.86 | 0.91 | 0.94 |
| NRS25 | 19 | 0 | 0.83 | 0.91 | 0.92 |
| NRS26 | 3 | 0 | 0.85 | 0.95 | 0.95 |
| NRS27 | 31 | 0 | 0.86 | 0.9 | 0.92 |
| NRS28 | 10 | 0 | 0.9 | 0.99 | 0.99 |
| NRS29 | 7 | 0 | 0.85 | 0.93 | 0.93 |
| NRS30 | 7 | 0 | 0.78 | 0.82 | 0.82 |

Abbreviations: Oph (years), number of years of experience as a board-certified ophthalmologist; Ret (years), number of years of experience as a retina specialist; RS, retina specialist; NRS, non-retina specialist; *No AI*, without AI assistance; *AI Prob*, AI assistance with probability score; *AI Prob+Evid*, AI assistance with probability score and visual evidence heatmap
